# Unlocking the Potential for Genetic Engineering of the Straw-Degrading Mushroom *Stropharia rugosoannulata* by Constructing a CRISPR/Cas9 Gene Editing System

**DOI:** 10.3390/jof12040269

**Published:** 2026-04-08

**Authors:** Haibo Hao, Shuzhen Song, Qian Wang, Zongjun Tong, Wen Xu, Jinxiao Yang, Yihong Yue, Tingting Xiao, Yuchen Zhang, Jinjing Zhang, Hui Chen

**Affiliations:** 1 National Research Center for Edible Fungi Biotechnology and Engineering, Key Laboratory of Applied Mycological Resources and Utilization, Ministry of Agriculture, Shanghai Key Laboratory of Agricultural Genetics and Breeding, Institute of Edible Fungi, Shanghai Academy of Agricultural Sciences, Shanghai 201403, China; 20230505@saas.sh.cn (H.H.); shu_zhen9@163.com (S.S.);; 2 College of Food Science and Technology, Shanghai Ocean University, Shanghai 201306, China; 3 Eternal Genediting (Beijing) Technology Co., Ltd., Beijing 201800, China

**Keywords:** *S. ugosoannulata*, CRISPR/Cas9, gene editing, *Agrobacterium*-mediated transformation, mutation efficiency

## Abstract

The artificially cultivated edible mushroom *Stropharia rugosoannulata* is widely promoted and cultivated in China because of its ability to efficiently decompose agricultural and forestry waste. However, methods for CRISPR/Cas9 genome editing have not yet been established for *S. rugosoannulata*. In this study, we identified three *SrU6* promoters in *S. rugosoannulata* and constructed the CRISPR/Cas9 expression vector GPiE-SrU6. Moreover, we found that mutant strains were obtained only when the expression of the single guide RNA (sgRNA) was driven by the *SrU6*-3 promoter. We subsequently employed a tandemly repeated SrU6-tRNA-sgRNA module to knock out two sites within the *ura3* gene. The expression vector was introduced into the mycelium via Agrobacterium-mediated transformation (ATMT). Following dual selection with 60 μg/mL hygromycin (Hyg) and 0.2 mg/mL 5-fluoroorotic acid (5-FOA), stable transformants were obtained and subcultured. The mutation efficiency at the targeted *ura3* locus was subsequently assessed. The CRISPR/Cas9 system successfully disrupted the target marker gene (*ura3*), achieving an editing efficiency of 14.9%. In summary, this study reports the first successful establishment of a CRISPR/Cas9 genome editing system in *S. rugosoannulata*. This study not only meets a future need for genetic manipulation tools for *S. rugosoannulata* but also provides a robust platform for engineering superior strains for eco-circular agriculture.

## 1. Introduction

*S. rugosoannulata* is a basidiomycete fungus whose fruiting body is rich in protein, dietary fiber, vitamins, and essential amino acids [1,2]. It also contains a variety of bioactive components, such as polyphenols, flavonoids, and ergosterol [3,4]. However, beyond its nutritional and medicinal merits, this basidiomycete has a unique and powerful ecological function: it acts as a robust “microbial cell factory” for agricultural waste conversion. Unlike other cultivated edible fungi, *S. rugosoannulata* decomposes straw without the need for suppressive fermentation or high-temperature sterilization. Its strong resistance to contaminants enables it to directly degrade agricultural and forestry waste in field or forest settings [5]. Despite this inherent potential, the natural degradation efficiency of wild-type (WT) strains often falls short of meeting the high demands for rapid agricultural waste disposal. To fully unleash its capability as a cell factory—for instance, by optimizing the expression of lignocellulolytic enzymes or rewiring metabolic flux for enhanced substrate utilization—sophisticated metabolic engineering is indispensable. However, the lack of precise genetic manipulation tools has long created a bottleneck for *S. rugosoannulata*, restricting genetic improvement to labor-intensive and imprecise traditional cross-breeding methods [6]. Therefore, establishing an efficient genome editing system is not merely a technical update but also a critical prerequisite for unlocking the metabolic engineering potential of this fungus.

Gene editing technology, particularly the clustered regularly interspaced short palindromic repeats and associated protein 9 (CRISPR/Cas9) system, offers the precision required to overcome traditional breeding and has ushered in a technological revolution in modern molecular breeding [7]. The CRISPR/Cas9 system has become a prominent tool, leveraged for its simple design, low cost, high efficiency, and ability to target multiple sites simultaneously [8]. It has since emerged as the dominant genome editing platform, following zinc finger nucleases (ZFNs) and transcription activator-like effector nucleases (TALENs) [9]. The sgRNA directs the Cas9 nuclease to a precise genomic location. Following protospacer adjacent motif (PAM) recognition, Cas9 cuts the DNA, leading to the formation of double-strand breaks (DSBs). Subsequently, the cell repairs the break via the error-prone nonhomologous end joining (NHEJ) pathway, which often results in insertions or deletions (indels) that can knock out gene function. Alternatively, if a homologous repair template is provided, precise gene replacement or insertion can be achieved through the homology-directed repair (HDR) pathway [10]. This technology has been successfully applied in various biological systems, including bacteria [11], fungi [12], and animals [13,14]. In plants, CRISPR/Cas9 technology has been widely applied to improve rice quality, primarily through targeted editing of starch branching enzyme-encoding genes (*SBEI* and *SBEIIb*) to develop varieties with high amylose content [15]. In filamentous fungi, CRISPR/Cas9 technology has been applied for metabolic engineering of various industrial strains, including *Aspergillus nidulans* [16] and *Aspergillus oryzae* [17]. This has significantly advanced the study of secondary metabolite biosynthesis pathways and related biological questions in these organisms.

As a distinctive group of filamentous fungi, basidiomycetes hold significant application value in the targeted breeding of fruiting body yield and bioactive components through the use of the CRISPR/Cas9 gene editing technology system. Currently, gene editing systems are being developed for economically important edible mushroom varieties. These include *Lentinula edodes* [18], *Flammulina filiformis* [19], *Pleurotus ostreatus* [20], *Pleurotus eryngii* [21], and *Ganoderma lucidum* [22]. Among these, CRISPR/Cas9-based studies on the growth, development, and secondary metabolite biosynthesis have been the most extensive in *G. lucidum* [23,24]. In the current development of genome editing systems, the *ura3*/*pryG* gene is frequently selected as a target. The endogenous *ura3* gene in fungi encodes orotidine-5′-phosphate decarboxylase (OMP decarboxylase), which catalyzes the conversion of orotidine-5′-phosphate (OMP) to uridine monophosphate (UMP). In the presence of 5-FOA, the functional OMP decarboxylase converts it into the toxic compound 5-fluorouracil, thus inhibiting cell growth. In contrast, *ura3* loss-of-function mutants are resistant to 5-FOA, enabling positive selection [25]. Therefore, the *ura3* gene is commonly targeted for use in genome editing as a positive selection marker for mutant screening. Additionally, other marker genes, such as those conferring resistance to hygromycin (*hph*) or carboxin (succinate dehydrogenase, *sdh*), are widely used for mutant selection [21,26]. In terms of editing methodology, some fungal systems utilize the delivery of preassembled sgRNA-Cas9 ribonucleoprotein (RNP) complexes. This enables editing via the NHEJ pathway and avoids the potential issue of exogenous DNA contamination [27,28]. Furthermore, base editing systems have been increasingly applied in plants, enabling precise nucleotide substitution without relying on DNA double-strand breaks [29]. However, their application in basidiomycete gene editing has not yet been reported.

Although CRISPR/Cas9 editing systems have been successfully established in numerous edible fungi, their application has not yet been extended to commercial varieties, likely due to concerns over the food safety of genetically modified organisms. Consequently, the development of gene-edited edible mushroom varieties, including the removal of selectable marker genes, requires further research. However, the potential of *S. rugosoannulata* extends beyond its traditional value as a food source. It also holds significant promise for development into an efficient engineering strain for straw degradation. Given the lack of a standard gene-editing system in *S. rugosoannulata*, we first identified an endogenous *SrU6* promoter suitable for sgRNA expression in this fungus. We then codon-optimized the Cas9 protein for basidiomycetes and employed ATMT, which enabled the efficient delivery of exogenous genes. To validate the efficiency of the gene-editing system, we knocked out the endogenous *ura3* gene. This work provides a technical foundation for future studies on fruiting body development, flavor compound biosynthesis, and substrate degradation mechanisms in *S. rugosoannulata*.

## 2. Materials and Methods

### 2.1. Strains and Cultivation

The monokaryotic strain Y27 of *S. rugosoannulata* used in this study was obtained through protoplast monokaryonization from strain DQ-1 (CGMCC5.2211), which is preserved at the China General Microbiological Culture Collection Center [5]. The monokaryotic strain Y27 of *S. rugosoannulata* (WT strain) was cultured on potato dextrose agar (PDA) medium (containing 200 g potato, 20 g sucrose, 20 g agar, and 1000 mL water) at 25 °C for experimental research; the screened uracil auxotrophic mutant strain was subsequently grown on PDAU (PDA medium supplemented with 5 mM uracil). Both the WT and the mutant strains were inoculated onto minimal medium (MM) (containing 20 g/L glucose, 1.5 g/L (NH_4_)_2_HPO_4_, 0.3 g/L MgSO_4_·7H_2_O, 1.0 g/L KH_2_PO_4_, 500 μg/L thiamine HCl, and 15 g/L agar) for growth and phenotypic observation. When inoculating the strains onto different culture media, three biological replicates were performed for each treatment. *Escherichia coli* TP10 and *A. tumefaciens* GV3101 were cultured in lysogeny broth (LB) medium (containing 10 g/L tryptone, 10 g/L NaCl, and 5 g/L yeast extract) supplemented with antibiotics (kanamycin and rifampicin) for vector construction and infection experiments.

### 2.2. Identification of Endogenous U6 Promoters

To identify the *SrU6* promoter in *S. rugosoannulata*, the nucleotide sequences of human U6 snRNA (NCBI: NR_004394.1) and the U6 snRNA gene of *A. nidulans* (GenBank: MH032752.1) were used as references. Homologous sequences were identified by performing a local BLAST search against the *S. rugosoannulata* genome using TBtools v2.056. Comparative analysis and alignment of the U6 promoter sequences from *Homo sapiens*, *G. lucidum*, *A. nidulans*, and *S. rugosoannulata* were then conducted with DNAMAN v10 software.

### 2.3. Plasmid Construction

In this study, the GPiE plasmid was used as the backbone, and the Cas9 gene sequence was synthesized based on the codon preference of basidiomycetes. The Cas9 sequence consists of 4101 deoxyribonucleotides. The Cas9 gene is driven by the constitutive promoter of the glyceraldehyde-3-phosphate dehydrogenase gene (Srgpd). The nucleotide sequence of *ura3* was uploaded to an online sgRNA design tool (http://grna.ctegd.uga.edu/ (accessed on 15 April 2025)), and only sequences with high scores and starting with a ‘G’ at the 5′ end were selected as candidate sgRNAs. In addition, the vector also contains a hygromycin resistance gene (*hyg*) expression module. All the primers used for vector construction are listed in Supplementary Table S1.

### 2.4. Construction of the ATMT System

The mycelium of *S. rugosoannulata*, precultured on PDA medium for 5 days, was inoculated into 100 mL of PDB medium in a shake flask and incubated at 25 °C with shaking at 150 rpm for 8 days. When the mycelial pellets reached a diameter of 2–4 mm, the mycelium was transferred to a sterilized blender and homogenized for 5 s, followed by collection using nonwoven fabric. The mycelium was rinsed once with sterile water and once with induction medium (IM) (containing 1% K buffer, 2% M-N solution, 1% CaCl_2_, 0.01% FeSO_4_, 20% NH_4_NO_3_, 50% glycerol, 4% MES, 50% glucose and 0.2% acetosyringone) to remove residual medium and then transferred into a sterile 50 mL centrifuge tube. Then, 5 mL of IM was added, and the mixture was incubated in the dark at 25 °C without shaking for 3–6 h. *Agrobacterium* was subsequently inoculated into LB medium supplemented with rifampicin and kanamycin and cultured with shaking at 200 rpm and 28 °C for 3 days. When the bacterial culture reached an OD_600_ of 0.5–0.8, it was transferred to a sterile 50 mL centrifuge tube and centrifuged at 9000 rpm and 4 °C for 5 min. The *Agrobacterium* cells were subsequently washed once with IM, resuspended in 5 mL of IM and incubated statically in the dark at 28 °C for 3–6 h. Finally, the precultured *S. rugosoannulata* mycelium and the *Agrobacterium* suspension were mixed together and cocultivated statically in the dark at 25 °C for 96 h.

### 2.5. Screening of the Optimal Drug Resistance Concentration in the Transformation System

To prevent *Agrobacterium* contamination after the completion of cocultivation, the *Agrobacterium* inhibitor cefotaxime sodium (Cef) was added to the selection medium. *S. rugosoannulata* was inoculated on PDA medium supplemented with 0, 200, 400, 600, or 800 mg/L Cef and cultured at 25 °C in the dark for 7 days. Phenotypic observations were then conducted to screen for an appropriate experimental concentration. To investigate the lethal concentration of Hyg, *S. rugosoannulata* was inoculated on PDA medium supplemented with 0, 20, 40, 60, or 80 μg/mL Hyg (Sangon Biotech Co., Ltd., Shanghai, China) and cultured under dark conditions at 25 °C for 7 days to observe the phenotypes and determine the appropriate experimental concentration. Furthermore, *S. rugosoannulata* was inoculated onto PDA resistance medium supplemented with 0, 0.05, 0.1, 0.2, or 0.3 mg/mL 5-FOA (Solarbio Science & Technology Co., Ltd., Beijing, China). The cultures were incubated at 25 °C in the dark for 7 days, after which the phenotypes were observed to determine the appropriate lethal concentration of 5-FOA. All of the above inoculation and culture experiments were performed with three biological replicates.

### 2.6. Screening and Identification of Mutant Strains

The cocultured mycelia were rinsed with sterile water containing Cef, repeatedly washed to remove the *Agrobacterium*, blotted dry, and then transferred to selective PDAU supplemented with 60 µg/mL Hyg and 600 µg/mL Cef. After 7–10 days of incubation, all the transformants grown on this medium were transferred to PDAU supplemented with 0.2 mg/mL 5-FOA and cultured for another 7 days. Transformants were picked and successively subcultured five times. Genomic DNA was then extracted from the transformants using a Fungal Genomic DNA Extraction Kit (Sangon Biotech Co., Ltd., Shanghai, China). The extracted DNA was stored at −20 °C for subsequent experiments.

Using high-fidelity DNA polymerase (Sangon Biotech Co., Ltd., Shanghai, China), PCR was performed with DNA from different transformants as templates to verify the presence of the hygromycin resistance marker in all the transformants, thereby confirming successful transformation. Furthermore, the *ura3* gene was amplified by PCR, chromatograms obtained after Sanger sequencing were analyzed with SuperDecode software_win (parameter: cutoff signal ratio = 0.3), and mutation types were precisely identified by comparing the WT and transformant chromatograms. Strains exhibiting insertions, substitutions, or deletions at the target site were identified as mutant strains [30]. In addition, we calculated the transformation efficiency by counting the edited strains among the total transformant population across three rounds of transformation.

### 2.7. Analysis of Mutant Strain Phenotype and Copy Number of Exogenous Gene Integration into the Genome

To assess the growth characteristics and nutritional requirements of the strains, the WT and mutant strains were inoculated on MM, PDAU, and PDAU+5-FOA media to observe the differences in growth phenotypes and to determine the effect of uracil on the growth of uracil auxotrophic strains generated by gene editing. Three biological replicates were performed for each treatment.

Using the genomic DNA of *S. rugosoannulata* as the internal reference gene standard, a 5-fold serial dilution was performed, and PCR amplification was carried out using 18S as the primer. Meanwhile, a plasmid containing the *hyg* gene was used as the exogenous gene standard, also subjected to a 5-fold serial dilution with the same dilution factor, and PCR amplification was performed using *hyg* as the primer. Finally, standard curves for real-time fluorescent quantitative PCR of the internal reference gene and the exogenous gene were generated. DNA was extracted from five randomly selected positive transgenic strains, and qPCR analysis was conducted for the 18S and *hyg* genes to calculate the copy number of the exogenous *hyg* gene inserted into the genome.

### 2.8. Genome-Wide Off-Target Prediction and Detection

For a selected region, targets with NGG PAM and a GC content of 30–70% were randomly chosen in accordance with conventional criteria. Off-target detection was performed using the CRISPR RGEN Tools according to the instructions for the portable version of Cas-OFFinder (http://www.rgenome.net/cas-offinder/portable (accessed on 15 April 2025)). Potential off-target sites with nucleotide mismatches were identified. Based on the genomic locations of these potential off-target sites, primers were designed. Each forward and reverse primer was constructed from 5′ to 3′ as follows: an adapter sequence (for library preparation and sequencing), a barcode sequence, and a target-specific amplification primer sequence (Supplementary Table S2). PCR amplification was then performed using 2× KeyPo Master Mix (Dye Plus) (Vazyme, Nanjing, China, PK511-01) to obtain PCR products. The PCR products carrying different barcode sequences were pooled and submitted to Tsingke Biotechnology Co., Ltd. (Beijing, China) for FastNGS sequencing. After obtaining the deep sequencing data, CRISPResso2 was used for analysis, and statistics were processed using Python 3.14 and Excel.

### 2.9. Statistical Analysis

Transformants were initially screened using antibiotic-containing medium and validated using hygromycin resistance markers. Further confirmation was performed by Sanger sequencing to identify mutant strains edited via the CRISPR/Cas9 system. All experiments were performed with three biological replicates. The editing efficiency was calculated by counting the edited strains among all transformants from the three transformation experiments. All experimental data obtained from the assays were analyzed and visualized using Excel and GraphPad 8.0.

## 3. Results

### 3.1. Analysis of SrU6 Promoters in S. rugosoannulata

**Figure 1:**
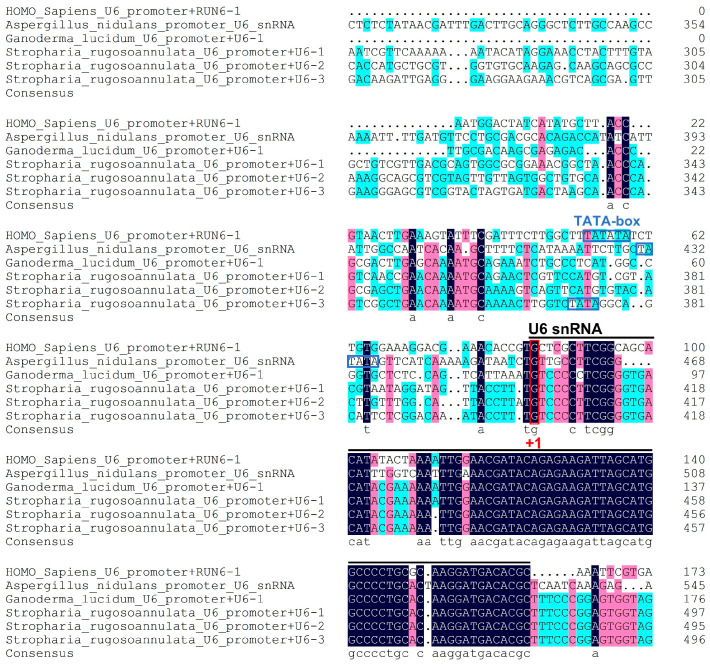
Analysis of U6 promoters in *S. rugosoannulata*. Multiple sequence alignment of *S. rugosoannulata* U6 small nuclear RNAs with those from *H. sapiens*, *A. nidulans*, and *G. lucidum*. The black line indicates the human U6 snRNA sequence. The conserved “TATA” box element is highlighted with a blue box. The nucleotide ‘G’ in the SrU6 promoter, recognized as the transcription start site (+1), is marked with a red box. Different colors in the alignment represent varying degrees of identity: nucleotides with 100% identity are shown in black, those with ≥80% identity are shown in dark red, and those with ≥60% identity are shown in light blue. The multiple sequence alignment was performed using DNAMAN software.

In this study, using the human U6 snRNA gene and the *A. nidulans* U6 snRNA gene as references, a homologous BLAST search was performed on the *S. rugosoannulata* genome, leading to the identification of three candidate genes, designated SrU6-1, SrU6-2, and SrU6-3. All these genes share conserved domains with RUN6-1 and U6 snRNA. Because the nucleotide “G” served as the transcription start site, we analyzed the SrU6 promoter sequences upstream of this site. The results revealed significant differences between the promoter regions of *S. rugosoannulata* and those of *H. sapiens*, *A. nidulans*, and *G. lucidum*. The “TATATA” box is present in both *H. sapiens* and *A. nidulans*. However, our analysis of the SrU6-1, SrU6-2, and SrU6-3 promoters indicates that the presence of a “TATA” box is exclusive to SrU6-3, a finding that represents a significant distinction (Figure 1). The sequences of all three candidate genes were more similar to that of *G. lucidum*, which may represent a distinctive characteristic of basidiomycete fungi.

### 3.2. Construction of Vectors for the CRISPR/Cas9 Gene Editing System

**Figure 2:**
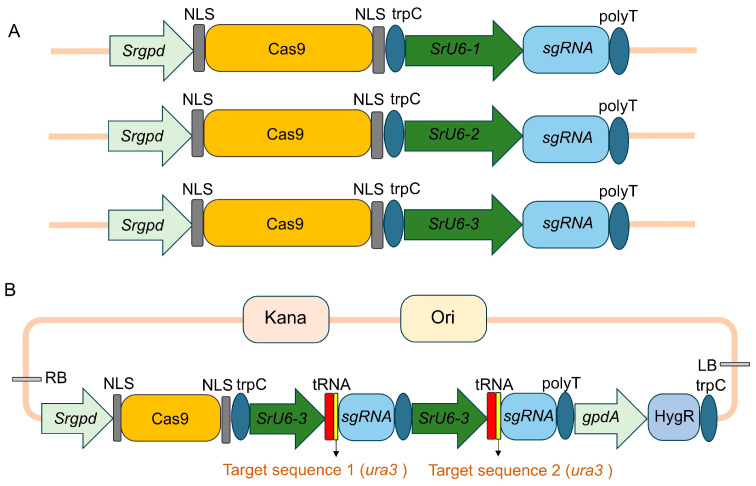
Schematic diagram of CRISPR/Cas9 gene editing vector construction. (**A**) Partial schematic diagram of sgRNA expression driven by the endogenous promoters SrU6-1, SrU6-2, and SrU6-3. (**B**) The GPiE-SrU6 expression plasmid includes a tRNA-sgRNA cassette driven by the *SrU6* promoter, Cas9 driven by the *Srgpd* promoter, *Hyg* driven by the *gpdA* promoter, and a terminator sequence.

With respect to the *S. rugosoannulata* genome, we analyzed and identified the *Srgpd* gene. A 2000 bp sequence upstream of the “ATG” start codon was selected as the *Srgpd* promoter to drive the expression of the Cas9 gene, which included two nuclear localization signals (NLSs) located at the 5′ and 3′ termini. Three 400 bp RNA polymerase III (pol III) promoters, SrU6-1, SrU6-2, and SrU6-3, were used to drive the expression of the sgRNAs. Additionally, a tRNA sequence was incorporated before the sgRNA target in tandem to enhance editing efficiency (Figure 2A). The validation results revealed that mutants were identified only in the GPiE-SrU6-3 transformants, whereas the other two promoters failed to produce any (Supplementary Table S3). Therefore, SrU6-3 was selected to drive the sgRNA. Furthermore, the GPiE-SrU6 vector contains two tandem “tRNA-sgRNA” expression modules, a *gpdA* promoter driving the hygromycin B resistance (*hyg*) selectable marker gene, and two editing sites targeting the *ura3* gene, for subsequent editing work in fungal hyphal cells (Figure 2B).

### 3.3. Establishment of the ATMT System and Screening of Antibiotic Concentrations

**Figure 3:**
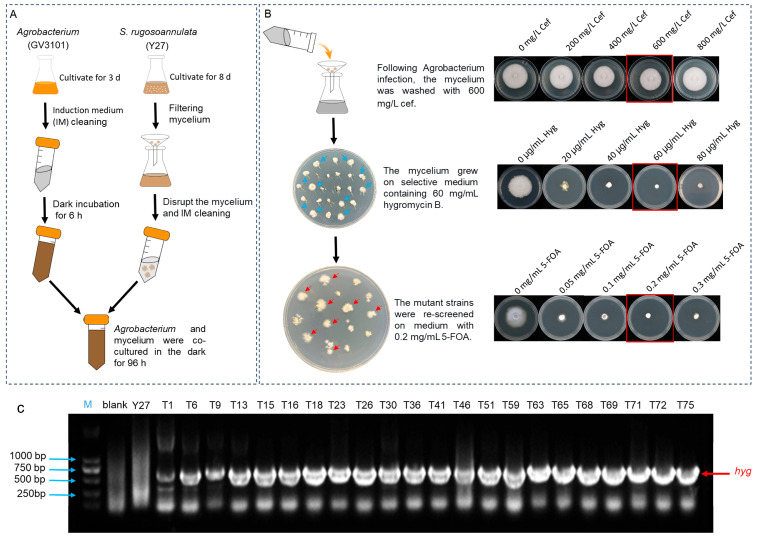
Establishment of an ATMT system for *S. rugosoannulata* mycelium. (**A**) The GPiE-SrU6 plasmid carrying two knockout targets was introduced into *A. tumefaciens* GV3101 for amplification, followed by cocultivation of wounded mycelium with *Agrobacterium* for 96 h. (**B**) After cocultivation, the mycelium was washed in medium containing 600 mg/L Cef, inoculated onto PDA medium supplemented with 60 µg/mL Hyg for primary screening, and subsequently transferred onto PDA medium containing 0.2 mg/mL 5-FOA for secondary screening. Growth on different selective drugs (Cef, Hyg and 5-FOA), with three biological replicates for each treatment. (**C**) Gel electrophoresis image of the hygromycin gene amplified from DNA extracted from some candidate transgenic strains.

To construct an ATMT system in *S. rugosoannulata*, we first transformed the constructed CRISPR/Cas9 vector plasmid into *A. tumefaciens* strain GV3101 and cultured it for amplification. Then, the cultured Y27 mycelium was wounded and cocultivated with the transformed *Agrobacterium* in IM (Figure 3A). Cefotaxime sodium is an effective inhibitor of *Agrobacterium* [31]. We found that even at concentrations as high as 800 mg/L, Cef had no inhibitory effect on the mycelial growth of *S. rugosoannulata*. To avoid potential stress on the mycelium caused by high antibiotic concentrations, we selected 600 mg/L Cef to suppress *Agrobacterium* growth. Concurrently, tolerance tests of the parental WT strain to different concentrations of Hyg and 5-FOA were conducted. The results demonstrated that treatment with 60 μg/mL Hyg and 0.2 mg/mL 5-FOA effectively inhibited the growth of the WT strain. Therefore, primary screening was performed on medium supplemented with 60 μg/mL Hyg (Figure 3B). DNA was subsequently extracted and verified via PCR targeting the *hyg* gene (Figure 3C). The obtained transgenic strains were then subjected to secondary screening on medium supplemented with 0.2 mg/mL 5-FOA (Figure 3). Viable transformants were picked for subculture. Furthermore, qPCR analysis confirmed the single-copy integration of the exogenous gene into the *S. rugosoannulata* genome, which was further verified to ensure stable expression of the exogenous gene (Supplementary Table S4).

### 3.4. Identification of Mutant Strains in Transformants and Off-Target Prediction

**Figure 4:**
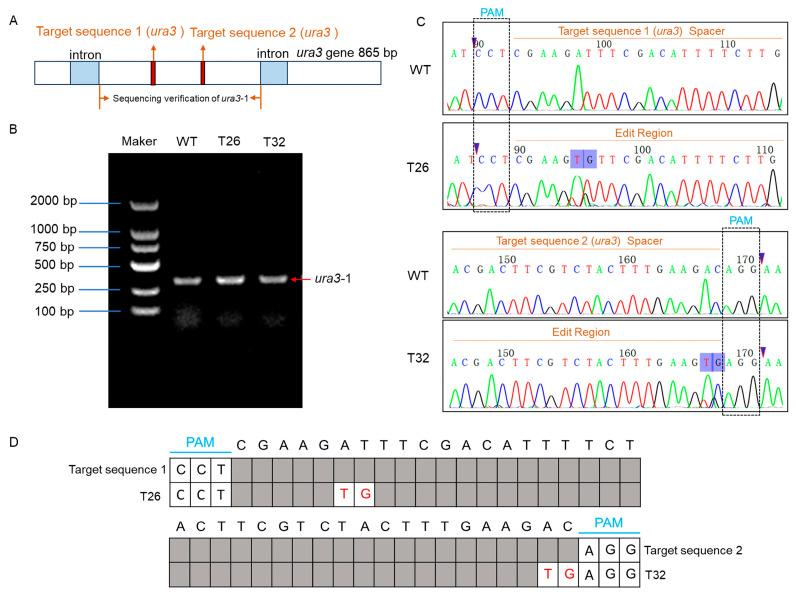
Disruption of the *ura3* gene target via different sgRNA sites in the CRISPR/Cas9 system. (**A**) Full-length structure of the *ura3* gene. (**B**) Electrophoresis diagram of PCR amplification of the partial ura3 gene from WT and mutant strains. (**C**) Sequencing peak maps of WT strains and mutant strains (T26 and T32) at target 1 and target 2. The black boxes indicate the PAM sequences. (**D**) Schematic diagram of mutation sites in the gene-edited strain.

The full length of the target gene *ura3* is 865 bp and contains two introns (Figure 4A). To verify whether the transformants were successfully edited, DNA was extracted from the transformants screened on 5-FOA medium after cultivation. Primers were then designed for the *ura3* gene to perform PCR, with the amplified region covering both target sites 1 and 2 (Figure 4B). The sequencing results indicate that in mutant strain T26, the sixth base “A” located upstream of the PAM sequence (CCT) at the first target site was substituted with “T”, and the seventh base “T” was substituted with “G”. Sequencing analysis revealed that in strain T32, at target site 2, the first base upstream of the PAM sequence (AGG) was substituted from “C” to “G”, and the second base was substituted from “A” to “T”. Therefore, these base substitutions may lead to the disruption of the *ura3* gene function.

Mismatches between PAM and sgRNA typically cause off-target effects. Whole-genome sequencing of *S. rugosoannulata* was performed as a reference database, and 20 potential off-target sites with 3–4 nucleotide mismatches were identified. Primers were designed for the genomic sequences of 8 randomly selected potential off-target sites. After amplification, FastNGS sequencing was performed, and no off-target events were detected (Supplementary Table S5). The predictive information not only demonstrates the high specificity of the gRNA in this study, but also establishes a foundation for future research on the safety of gene editing in *S. rugosoannulata*.

### 3.5. Analysis of the Growth Phenotypes and Editing Efficiencies of Edited Strains of S. rugosoannulata

**Figure 5:**
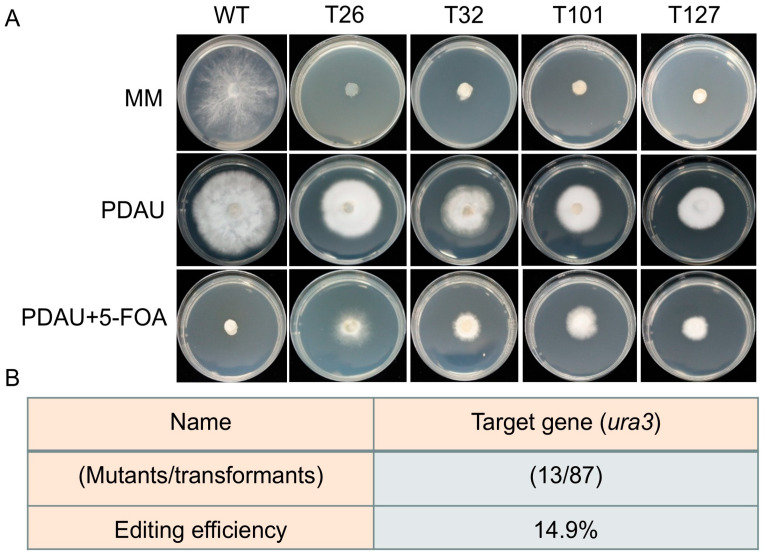
Growth phenotypes of mutant strains on different culture media and editing efficiency of the *ura3* gene. (**A**) Growth phenotypes of WT and mutant strains on MM, PDAU (PDA medium supplemented with 5 mM uracil), and PDAU+5-FOA (PDAU supplemented with 0.2 mg/mL 5-FOA). The WT and mutant strains were grown on different culture media, with three biological replicates per treatment. (**B**) Analysis of the editing efficiency of the *ura3* gene in *S. rugosoannulata* using the CRISPR/Cas9 system.

We then inoculated the WT and edited strains (T26, T32, T101 and T127) on MM. The results revealed that although the growth of the mutant strains was not completely lethal, their growth was almost completely inhibited. However, when the mutant strains were inoculated on PDA medium supplemented with uracil (PDAU), their growth was restored, although the growth phenotype was significantly weaker than that of the WT strain (Figure 5A). These findings confirm that the mutations generated by CRISPR/Cas9 successfully disrupted the function of the *ura3* gene, resulting in uracil auxotrophic mutants. Additionally, we calculated the editing efficiency based on 87 transformants. The constructed CRISPR/Cas9 system achieved an editing efficiency of 14.9% for the *ura3* gene (Figure 5B). In summary, the CRISPR/Cas9 gene editing system we established can effectively facilitate the mutation of endogenous *ura3* gene in *S. rugosoannulata*.

## 4. Discussion

*S. rugosoannulata* is cultivated primarily using agricultural crop straw for growth and is promoted for cultivation during the winter fallow period in the rice-growing areas of the middle and lower reaches of the Yangtze River Basin. Unlike typical straw-decaying fungi such as Agaricus bisporus and Volvariella volvacea, which require the cultivation substrate to be composted, *S. rugosoannulata* can directly degrade straw into organic fertilizer for in situ return to fields or forests [5,32]. In recent years, it has been promoted as a key variety for efficient straw decomposition to address the challenges associated with straw disposal in agricultural systems [32]. Therefore, beyond its edible value, utilizing it as an engineering strain for highly efficient straw degradation remains important. In this study, the cultivated variety “Huqiu No. 1” (DQ-1) strain from the Shanghai region was subjected to monokaryonization to obtain the “Y27” strain. Using this strain, a CRISPR/Cas9-mediated gene editing system was established. By optimizing the endogenous SrU6 promoter and constructing a tRNA-sgRNA module, multiple base substitutions were successfully introduced at two target sites of the *ura3* gene in *S. rugosoannulata*, thereby generating gene-edited mutant strains.

Currently, genetic transformation in filamentous fungi primarily involves two methods: PEG-mediated protoplast transformation and ATMT-mediated infection of mycelia [33]. This study primarily employed Agrobacterium-mediated transformation to introduce gene-editing plasmids into mycelial cells. For the antibiotic concentration screening in the transformation system, we found that 600 mg/L Cef was suitable as the inhibitory concentration for *Agrobacterium*, whereas 60 µg/mL Hyg was the optimal experimental concentration. Previous studies have shown that the concentration of hygromycin used in the ATMT system for the *S. rugosoannulata* HC7 strain was 50 µg/mL [34]. Therefore, the higher hygromycin concentration used in our study represents a more stringent screening condition. Additionally, 0.2 mg/mL 5-FOA inhibited the growth of *S. rugosoannulata*, whereas 0.4 mg/mL 5-FOA inhibited the growth of *G. lucidum* [35]. This difference may be related to variations in the tolerance of different fungi to 5-FOA.

The selection of appropriate gene target sites facilitates the evaluation of editing efficiency. Previous studies have shown that mutations in the pksP and lae1 genes in *A. fumigatus* [36] and *Trichoderma reesei* [27] can block pigment production, thereby facilitating the screening of mutants. Moreover, mutation of the sdh gene in G. lucidum and *Pleurotus ostreatus* can block ATP synthesis and confer resistance to carboxin [37,38]. In most basidiomycetes, such as *G. lucidum* [24], *F. filiformis* [39], *P. eryngii* [40], and *P. ostreatus* [20], the *ura3*/*pyrG* gene has been used as a target to establish editing systems. The loss of function of *ura3*/*pyrG* interrupts the uracil biosynthesis pathway and confers resistance to 5-FOA. This study also targeted the *ura3* gene and successfully achieved base mutations at distinct positions across two target sites. In the future, the engineered endogenous *ura3* mutant strain could serve as a chassis for developing antibiotic marker-free methods for functional gene knockout. This will enable the development of antibiotic marker-free methods for functional gene knockout, using the created endogenous *ura3* mutant strain as a chassis.

In animals and plants, several strategies for expressing multiple sgRNAs, such as Pol III promoter: sgRNA tandem repeats [41], the endogenous tRNA-processing system [42], hepatitis delta virus (HDV) and hammerhead (HH) ribozymes [43], have been reported. To improve gene editing efficiency, three endogenous *SrU6* promoters were identified. Notably, among the three *SrU6* promoters, only *SrU6-3* contains a canonical “TATA” box; this element was not found in the other two promoters. Previous studies have shown that the *U6* promoters in *G. lucidum* and *F. filiformis* also lack a canonical “TATA” box [19,24].

The “TATA” box is a key element of eukaryotic promoters and determines the selection of the transcription start site. Transcription can only begin after RNA polymerase is firmly bound to the “TATA” box [44]. The “TATA” box located 26 bp upstream of the transcription start site may be more prone to causing gene mutations after gene editing, thereby resulting in higher editing efficiency of the sgRNA driven by SrU6-3. Therefore, whether gene editing efficiency is associated with the presence of a “TATA” box requires further investigation. Additionally, we constructed a tandem array of endogenous SrU6-tRNA expression cassettes in the vector. In Cordyceps militaris, it has also been reported that RNA Pol III promoters and the chimeric AfU6-tRNAGly facilitate enhanced editing efficiency [45]. Therefore, our utilization of the SrU6-tRNA tandem array to drive sgRNA expression should contribute to enhanced editing efficiency. The delivery method of fungal gene editing systems depends on the application scenario and cell type, primarily comprising two approaches: one involves constructing components such as Cas9 and sgRNA into plasmids for delivery, while the other involves directly delivering in vitro assembled Cas9/sgRNA complexes [46]. Plasmid delivery can be achieved through either PEG-mediated transformation or ATMT, whereas RNP complex delivery typically relies on PEG-mediated transformation. Currently, RNP complexes face challenges such as large molecular assemblies, including difficulty crossing cell membranes and susceptibility to rapid clearance and degradation in vivo. However, studies have shown that Triton X-100 can expand the pores in protoplast membranes, thereby increasing the mutation acquisition rate under PEG-mediated transformation [35]. The advantage of ATMT lies in its low cost and the long-term persistence of the plasmid within the nucleus, enabling sustained production of Cas9 and sgRNA. This makes it well suited for applications requiring continuous editing or editing across multiple cell cycles. In this study, the ATMT technique was employed to integrate the editing plasmid into the genome of *S. rugosoannulata*. After screening, 87 transformants were obtained, and the final results indicated an editing efficiency of 14.9%. Although the editing efficiency was not high, this work has nonetheless broadened the application of CRISPR/Cas9 technology in fungi. In the future, the technical editing system for RNP delivery can be further optimized to achieve rapid, safe, and efficient gene editing by transiently expressing the Cas9 protein without integrating it into the genome of edible fungi. Furthermore, achieving marker-free editing or eliminating resistance markers is the ultimate goal of our gene-editing breeding program, as only then can we develop safe, transgene-free commercial varieties of edible fungi.

## 5. Conclusions

In summary, we have established a CRISPR/Cas9 system in *S. rugosoannulata*, which paves the way for its future application in basidiomycetes despite the current *ura3* editing efficiency of 14.9%. Future efforts will focus on enhancing this efficiency and leveraging the system for molecular breeding.

## Data Availability

The authors confirm that the data supporting the findings of this study are available within the article and its Supplementary Materials.

## Supplementary Materials

The following supporting information can be downloaded at: https://www.mdpi.com/article/10.3390/jof12040269/s1, Supplementary Table S1. Primer sequences intended for the construction of the GPiE-SrU6 plasmid and for sequencing verification of mutants; Supplementary Table S2. Primer sequences for off-target site detection in gene editing; Supplementary Table S3. Three distinct SrU6 promoters were utilized to individually drive sgRNA expression for screening the optimal promoter for gene editing; Supplementary Table S4. Copy number analysis of the *hyg* gene in transgenic *S. rugosoannulata*; Supplementary Table S5. Detection and analysis of off-target sites for gene editing in *S. rugosoannulata*.
